# Classification of Companion Diagnostics: A New Framework for Biomarker-Driven Patient Selection

**DOI:** 10.1007/s43441-021-00352-2

**Published:** 2021-11-28

**Authors:** Cynthia Huber, Tim Friede, Julia Stingl, Norbert Benda

**Affiliations:** 1 Department of Medical Statistics, University Medical Center Göttingen, Humboldtallee 32, 37073 Göttingen, Germany; 2 Faculty of Medicine, RWTH Aachen University, Aachen, Germany; 3 Research Department, Federal Institute for Drugs and Medical Devices, Bonn, Germany

**Keywords:** Predictive, Prognostic, Biomarker, Companion diagnostic, Classification, Patient selection

## Abstract

**Background:**

Modern personalized medicine strategies builds on therapy companion diagnostics to stratify patients into subgroups with differential benefit/risk. In general, stratification for drug response implies a treatment-by-subgroup interaction. This interaction is usually suggested by the drug’s mechanism of action and investigated in pharmacological research or in clinical studies. In these candidate genes or pathway approaches, either biological reasons for a differential benefit/risk or statistical interaction regarding a pharmacological or clinical endpoint or both may be given. For successful drug approval, demonstration of a positive benefit/risk balance in the intended patient population is required. This also applies to situations with biomarker-selected populations. However, further regulatory considerations relate to the usefulness and plausibility of the selected patients and benefit/risk extrapolations or alternative therapy options in biomarker-negative populations.

**Methods:**

To facilitate the specification of regulatory requirements and support the design of clinical development programmes, a systematic classification of biomarker-drug pairs is needed, in particular with regard to the expected underlying molecular mechanism and the clinical evidence.

**Results:**

A classification of five biomarker-drug categories is proposed related to increasing evidence on the biomarker’s predictive value in relation to a specific drug. We classified biomarkers into five ascending categories with increasing evidence on the predictive nature of the biomarker in relation to a specific drug according to the comparative pharmacological and clinical evidence.

**Conclusions:**

The proposed classification will facilitate regulatory decision-making and support drug development with respect to biomarker-related subgrouping, both, during clinical programme and at the time of marketing authorization application, since the grade of evidence on the differential power of the biomarker can be considered as an indicator for the usefulness of a biomarker-related subgrouping.

## Introduction

Precision medicine offers a promising vision on the development of new targeted drugs, in particular in areas with a high medical need. Assuming that some drugs may act differently in different patients, precision medicine is searching for a relevant interaction between patient and treatment resulting in an improved efficacy or tolerability in a given subgroup of patients. Whereas the expectations regarding tailor made medicines are high in many therapeutic areas, essentially only cancer drugs have been successfully approved yet in biomarker-defined subgroups [1, 2]. Advances in genomic sequencing increasingly lead to the investigation of genetic profiles used for stratifying patients to treatment response. In contrast to the high expectations in precision medicine, the usefulness of the biomarker-based selection of patients with respect to clinical outcomes is often not substantiated by clinical data and expectations may be too optimistic.

The investigation of a predictive biomarker profile assumes a positive treatment-by-subgroup interaction, i.e. a differential treatment effect in subgroups allowing for discriminating patient groups with different expected treatment effects. Predictive biomarkers should be capable to discriminate subgroups with different expected treatment effects related either to an efficacy or to a safety outcome, whereas prognostic biomarkers should indicate a favourable or unfavourable natural course of the disease [3–5]. In the context of personalized therapy, the focus is on predictive biomarkers whereas purely prognostic biomarkers are not of primary interest.

The treatment-by-subgroup interaction is usually driven by pharmacological drug action and investigated in pre-clinical research or in surrogate endpoints in clinical studies suggesting that biological reasons for differential benefit/risk and statistical interactions are interrelated. Demonstration of a true (and relevant) interaction with respect to a clinically relevant endpoint as part of a confirmatory analysis can be either done by a prespecified subgroup analyses or by a statistical model including treatment-by-subgroup interactions [6]. This, however, often remains a difficult task as the required sample sizes are often too large. Furthermore, an interaction on a specific statistical scale does not necessarily imply that there is a biological interaction but may just be induced by the choice of the scale.

Successful drug approval usually needs demonstration of the effectiveness and tolerability in the biomarker-defined subgroup but does not necessarily need a proof of the usefulness of the restriction to a limited population. However, in drugs that have been approved in a biomarker-restricted population, extrapolation of benefit risk to the biomarker-negative population may apply later on, and enlarge the indicated patient population for which a drug is authorized. In this case, heterogeneity analyses of the overall population need to be done, and data in biomarker-negative persons are required. In case a drug has been approved in a biomarker-restricted population only, and the mode of action of a drug-biomarker combination makes it unlikely that biomarker-negative persons will have a positive benefit/risk, it is unnecessary, and may be even unethical to demand data from treatment in biomarker-negative subjects. Thus, evidence of a truly predictive biomarker that is capable to discriminate patients that will benefit from treatment from those who do not is important. However, such evidence is often scarce, because clinical trials are usually not powered to detect a statistically significant treatment-by-subpopulation interaction, which is further complicated by additional sources of variability, see, e.g. [7, 8].

Extrapolation of the benefit-risk balance to the biomarker-negative population largely depends on the level of biological plausibility of the biomarker with respect to its predictivity regarding a given medicine. Biological plausibility together with the empirical evidence generated by clinical or non-clinical studies, both, define the usefulness of the biomarker with respect to stratified medicine. Since specific regulatory considerations relate to this selection of patient subgroups, the usefulness and discriminative value of a supposedly predictive biomarker is of regulatory interest as well. To facilitate the specification of regulatory requirements and to support the design of clinical development programmes, a classification of biomarkers is needed, that is capable to properly separate between empirical evidence versus biological plausibility, and allow a decision towards extrapolation of the benefit-risk balance to the biomarker-negative population. Available biomarker classifications just distinguish between prognostic (forecasting the course of the disease), predictive (predicting a differential treatment effect) and surrogate endpoints (intended to replace a clinical endpoint) [9–11]. The categories proposed by the FDA [12] additionally differ between diagnostic, monitoring, safety, susceptibility and pharmacodynamic biomarkers. Classifications of biomarker-drug pairs proposed by PharmGKB [13] and Vivot et al. [14] refer to the guidance for genetic testing based on the drug label and do not take the type of presented evidence supporting the type of biomarker (predictive/prognostic) into account.

Tsourounis et al. [1] discuss new options to develop diagnostic tools and new treatments for cancer, genetic disorders, and infectious diseases that may be particularly effective in biomarker-defined subpopulations, and discuss the key developmental and regulatory challenges and the importance of related regulatory and policy considerations. Ansari [15] presents an overview of the changing regulatory landscape for companion diagnostics through predictive biomarkers along with the challenges associated with developing a successful global regulatory strategy for a companion diagnostic. Enzmann et al. [16] describe the EMA’s principles for the assessment of the impact of companion diagnostics on the benefit–risk balance of medicines.

Patient subgroups are often defined based on biomarkers; the subgroup serves various purposes including improved definition of the disease or prognosis, exclusion of patients at increased risk of harm, prediction of beneficial drug response [17]*. However,* the evidence justifying the selection of patients based on specific biomarkers is highly heterogeneous. Within the scientific advice procedures at EMA, specific procedures are dedicated to support the qualification of innovative development methods including qualification of new biomarkers, see, e.g. [18] for novel biomarkers in Alzheimer disease. This procedure as well as FDA’s biomarker qualification programme offer the possibility to drug developers to discuss new biomarkers with respect to different context of use. For qualified biomarkers “specific interpretation and application in drug development and regulatory decision-making” can be relied upon within the stated context of use during drug development [19]. Although a specific interest lies in the capability of the biomarker to define a relevant patient population to be treated, the main focus of biomarker qualification is often on the prognostic value of a biomarker. In contrast, evidence for the predictive value of a biomarker, although more relevant to the indication of the related drug, is often sparse or unclear. Sources of this evidence are diverse, its relevance may be vague and a clear classification of the degree of evidence is missing.

Therefore, a framework is needed that allows for a reasonable classification of the underlying evidence resulting in regulatory and scientific recommendations for biomarker-based selection.

In this paper we propose a framework that allows for a classification according to the available evidence with respect to the biomarker’s capability to select subpopulations in which the benefit (or benefit-risk) of a given treatment is expected to be considerably superior to that in the complementary group. To illustrate the proposed classification and the available evidence in successful past approvals, we give examples for each category of biomarker-related drug approvals. The different classification groups should support regulatory decisions on clinical study/evidence of treatment data in biomarker-negative patients. The consequence of a biomarker test could then be used (1) to exclude biomarker-negative patients from treatment, (2) to stratify treatment to different dosing/ combination treatments/alternatives, (3) to be aware of an altered safety/efficacy risk in the biomarker-negative populations (as an example, resulting in a warning information in the Summary of Product Characteristics (SmPC)). It is further suggested that the use of promising biomarkers in precision medicine is not self-evident and that methodological effort is needed to substantiate the usefulness of a biomarker in the presence of limited clinical databases.

## Methods

The development of the classification scheme involves the following aspects: the evidence for predictive and/or prognostic biomarkers, the data type and the need for dichotomization, the scale of the effect size and the type of outcome related to the differential treatment effect.

### Essential Aspects for Biomarker Classification

#### Data Type and Dichotomization

A prognostic or predictive biomarker may be a binary, categorical or continuous variable. Often an originally continuous outcome is dichotomized to split the overall patient population. Dichotomization and specification of the cut-off value used are relevant for the size of the differential treatment in the resulting biomarker-defined subpopulations.

#### Predictive and/or Prognostic Biomarker

**Fig. 1:**
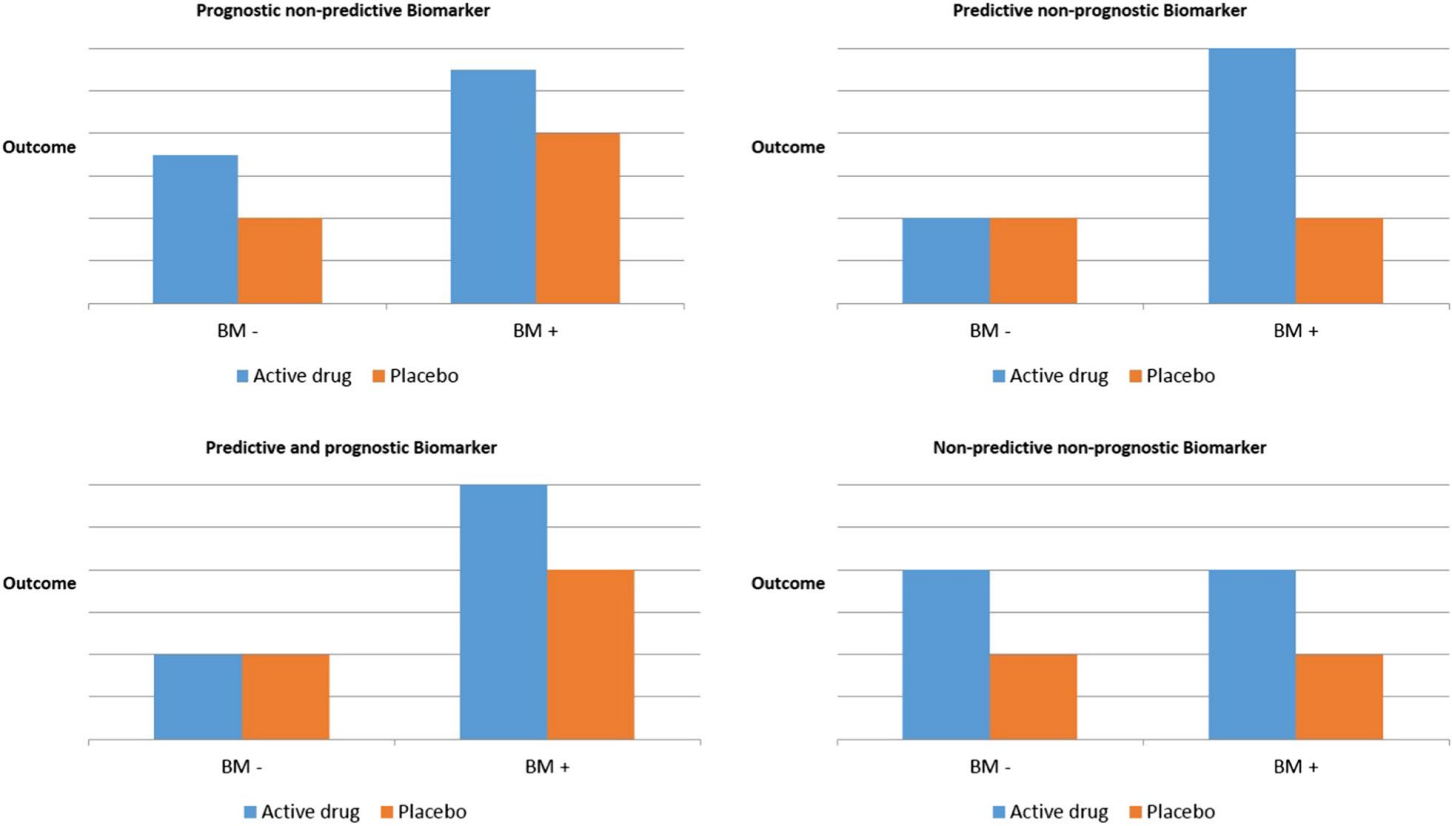
Schematic description of prognostic and predictive biomarkers related to differential treatment outcome. *BM*+ denotes biomarker-positive patients and *BM− *refers to biomarker-negative patients

A predictive biomarker predicts the response or lack of response to a particular treatment relative to another available treatment: Patients with specific values of the biomarker are expected to profit from the treatment more than others, i.e. the expected effect difference to a control in these patients is larger or smaller than in the others. A predictive biomarker is measured to determine the best treatment prior to the start of treatment. In statistical terms, a predictive biomarker implies a non-negative interaction between biomarker and drug. In contrast, a prognostic biomarker forecasts the course of the disease. Figure 1 shows how prognostic and predictive biomarker differ regarding the outcome in biomarker-positive patients (BM+) and its complementary group (BM−).

Example for evidence on a predictive role of a biomarker: Gefitinib (IRESSA) was approved in the EU in 2009. In the initial application, the applicant had submitted a marketing authorisation application (MAA) for the following indication: *IRESSA (gefitinib) for the treatment of adults with locally advanced or metastatic non-small cell lung cancer (NSCLC) eligible for further chemotherapy after receiving prior platinum based chemotherapy* [19].

Corresponding to the proposed indication, progression free survival was significantly improved in the overall population (HR = 0.74, 95% CI (0.65, 0.85), *p* < 0.0001) [20].

However, following the assessment and objections raised by the CHMP, a new indication was proposed: *IRESSA is indicated for the treatment of adult patients with locally advanced or metastatic non-small cell lung cancer (NSCLC) with activating mutations of EGFR-TK* [21]

In a clinical study (IPASS), treatment effect sizes with respect to objective response rate as well as progression free survival were clearly different in EGFR mutated patients as compared to EGFR non-mutated patients. Whereas a positive effect compared to Paclitaxel was shown for EGFR mutations, a negative effect was seen in non-mutated patients. Although this result was obtained in post hoc analysis where the majority of the patients could not be classified with respect to EGFR mutation, the result suggested a clear interaction between treatment and biomarker, though much less important in overall survival, see Table 1.

**Table 1:** Example: Results of the IPASS Study comparing Gifitinib (Iressa) vs carboplatin–paclitaxel in selected NSCLC patients [10]

Endpoint	Subgroup	Effect estimate	95% CI
Objective response rate	EGRF non-mutated (*n* = 176)	OR = 0.04	(0.005, 0.273)
	EGRF mutated (*n* = 261)	OR = 2.75	(1.646, 4.596)
PFS	EGRF non-mutated (*n* = 176)	HR = 2.85	(2.05; 3.98)
	EGRF mutated (*n* = 261)	HR = 0.48	(0.36; 0.64)
Overall survival	EGRF non-mutated (*n* = 176)	HR = 1.18	(0.86, 1.63)
	EGRF mutated (*n* = 276)	HR = 1.00	(0.76, 1.33)

*PFS* denotes Progression free survival, *HR* refers to the Hazard Ratio and *OR* to Odds Ratio

Hence, EGFR mutation can be regarded as a predictive biomarker with respect to Gifitinib and the endpoints ORR and PFS with highly significant results on the corresponding interaction. Accordingly, the indication of IRESSA was restricted to adult patients with locally advanced or metastatic non-small cell lung cancer (NSCLC) with activating mutations of EGFR tyrosine kinase.

#### Treatment Effect Scale

The scale on which the effects are evaluated matters and may lead to different conclusions: e.g., an effect may be given either as a difference in response probability (risk difference) or as the corresponding odds ratio. A prognostic biomarker would lead to an increased response probability in both study arms (verum or placebo) in the biomarker-selected subpopulation (BM+). Whether the effect difference of the active drug compared to control is larger in BM+ depends, however, on the scale used, as illustrated in the hypothetical example in Table 2, where no interaction is given for the risk difference, but a positive for the odds ratio [21]. More generally, if a positive treatment effect is given, the absence of interaction on one scale usually implies a non-zero interaction on the other.

**Table 2:** Hypothetical example: Scale dependency of predictive biomarker selection given a prognostic biomarker. Biomarker is predictive w.r.t. the odds ratio but not w.r.t. the risk difference

Subgroup	Response probability (placebo)	Response probability (verum)	Risk difference	Odds ratio
BM−	0.50	0.65	0.15	1.86
BM+	0.75	0.90	0.15	3.00

It is therefore important to base the assessment of the biomarker-drug pair on a specific scale that is considered most relevant.

### Type of Outcome Related to the Biomarker’s Predictivity: Efficacy/Safety/Exposure

#### Efficacy

In this category it is assumed that the biomarker is linked to specific efficacy measures but not to the tolerability of the drug. I.e. an interaction between drug and (dichotomized) biomarker is given for a relevant efficacy parameter.

#### Safety

In this category the biomarker is linked to specific safety measures but not to the efficacy of the drug. I.e. an interaction between drug and (dichotomized) biomarker is given for tolerability/safety (involving a different risk ratio in both subgroups).

#### Metabolism, Efficacy and Safety

In this category the biomarker is linked to both efficacy and tolerability of the drug, especially in those settings where the biomarker measures the metabolic activity, and biomarker-positive patients are expected to undergo a higher drug exposition than others potentially leading to a higher efficacy but also to a lower tolerability. In these cases, dose may be adjusted according to the categorization by the biomarker. Full dichotomization may not necessarily be useful in these cases.

## Results

### Evidence Based Biomarker-Drug Classification

In general, there is a need to differentiate biomarkers that are purely prognostic from those that are predictive since a prognostic marker would not necessarily justify that a part of the patients are not treated with the new treatment. Also given the difficulties to fully investigate the interaction between biomarker and treatment with respect to the relevant clinical outcome at least in a medium-sized clinical trial as described in  “Introduction” section, it appears paramount to classify a biomarker-drug pair with respect to the biomarker’s capability to properly identify the drug target preferably prior to large clinical investigations, see, e.g. [5]. A clinical trial that is powered to detect an interaction in a clinical endpoint indicating a predictive biomarker would usually be rather large unless the interaction is extremely large.

The usefulness of a biomarker for a given drug has to be assessed with respect to the biological plausibility relating to the pathogenic process and the drug’s mechanism of action and the empirical evidence determining the capability to predict a certain drug effect. Hence, two dimensions should be considered in a classification of biomarker-drug pairs. The first dimension concerns the mechanism of the biomarker measuring the drug’s action, i.e. the biological plausibility of a biomarker acting directly in the drug target. The second aspect concerns the precision of the biomarker in predicting the clinical outcome in drug efficacy or safety, regardless of the pharmacological mechanism behind.

Therefore, we propose to classify biomarker-drug pairs according to the pharmacological mechanisms and biological plausibility on one hand and the evidence with respect to clinical data on the other. In both cases the evidence is called *comparative*, if conclusions can be made whether the treatment benefit is differential with respect to the biomarker-related selection, which is related to the predictive value of the biomarker. Evidence may be derived, e.g., from a comparative animal or clinical study where a specific drug is compared to a control or relate to the specific drug action or target. If, e.g., a biomarker characterizes the drug target, it may be assumed that biomarker-positive patients benefit more than biomarker-negative patients. If the mechanism of action indicates that biomarker-negative patients may be harmed by the drug, ethical considerations might prevent the inclusion of biomarker-negative patients into clinical studies. If comparative evidence is given, the biomarker is specific to a given drug. Clinical evidence is considered to be comparative if clinical data are given that allow for evaluating the interaction between drug and biomarker.

In that sense, we propose the following higher-level classification:

*A: biomarkers with non-comparative pharmacological evidence only*

These are biomarkers where pharmacological mechanisms indicate a prognostic value, but that is unrelated to the drug target. Clinical investigations on any predictive value are not available. Nevertheless, indication of a prognostic value may qualify these biomarkers as candidates for predictive biomarkers requiring further investigations regarding the pharmacological effect in BM negative and BM positive patients.

This category includes phenotypes of drug action mechanism that may not be causally linked to the pathogenetic process, usually because of incomplete knowledge about the molecular mechanisms that link the biological effect of the drug with response. It applies to biomarkers that measure the effect of the drug on their biological substrate, where the causal relationship between this substrate and response is unknown or uncertain. A weak causal link between molecular drug action and response may be suggested. It includes phenotypes whose association with response may exclusively be of statistical nature. These biomarkers may still be predictive, but the mechanism at the basis of this prediction is essentially unknown.

*B: biomarkers with comparative clinical data only*

In this category comparative clinical data are available but with respect to the knowledge about the molecular mechanisms its characterization is similar to those in group A. With other words, unless a well conducted large trial stratified in BM positive and negative patients has demonstrated a significant interaction, a chance finding regarding the biomarker’s predictivity cannot be excluded. It appears highly unrealistic that a biomarker without sufficient information on its causal link to molecular drug action is investigated in a large clinical trial and shown to be predictive.

*C: biomarkers with comparative pharmacological evidence*

These biomarkers are to be characterized based on the phenotype’s capability to identify the causal mechanism that is responsible for therapy. Within this group, biomarker phenotypes may be classified into several groups, characterized by decreasing degrees of identification of a causal link between the phenotype and response.

This group consists of phenotypes that measure a pathogenetic process, such as a molecular mechanism of pathology, on which the drug directly acts. This phenotype is causally most specific, since it detects changes in the mechanism that is itself contributing to the disease pathology.

This group provides reasonable candidates for further investigation in subsequent clinical trials or for further analysis using data from existing trials. However, in cases where pharmacologic evidence suggests negative effects on patients, clinical evidence (as requested in the following levels) of a differential treatment effect in subgroups becomes unnecessary or impossible because of ethical concerns.

*D: biomarkers with comparative pharmacological and clinical evidence*

These biomarkers are to be characterized by the evidence described for group C and for which clinical data have been generated that allow for evaluating a differential effect in BM+ and BM− patients.

This is related to the predictive value of the biomarker. Evidence may be given, e.g., from a comparative animal or clinical study where a specific drug is compared to a control or relate to the specific drug action or target. If, e.g., a biomarker characterizes the drug target, it may be assumed that biomarker-positive patients benefit more than biomarker-negative patients. If comparative evidence is given, the biomarker is specific to a given drug.

*E: biomarkers demonstrated to be predictive in confirmatory clinical studies*

This group relates to the biomarker-drug pairs from group D for which a confirmatory clinical trial stratified in BM positive and negative patients has demonstrated a significant and relevant interaction between biomarker selection and treatment. For this group highest evidence for the usefulness of the biomarker would be given. It would require a pre-planned confirmatory analysis strategy. However, due to the fact, that sample sizes that exceed usual clinical trials are required to detect at least moderate interaction sizes, biomarker-drug pairs falling into this category are expected to remain scarce.

### Types of Biomarker Predictivity

For biomarker-drug pairs from category C, D and E several aspects like the pathogenetic mechanism, biomarker-efficacy and biomarker-safety relationship should be considered. Here we can distinguish three cases. For each case, we present examples of biomarker-drug pairs with comparative evidence in Table 3.

- The first example refers to a molecular change that is indicative for pathology and is also the direct drug target (e.g. BCR-Abl). The drug can act only in cells in which the molecular change assessed by the biomarker is present. Therefore, the efficacy is zero in biomarker-negative patients.
- The second example refers to a biomarker measures a specific pathway activation (e.g. driver mutation in tumour) but the efficacy finally depends on other surrounding factors like tumour heterogeneity or context of driver mutation (additional mutations downstream). In this case, drug response may depend not only on biomarker detection, and the biomarker may not be valid in all contexts (reduced biomarker specificity): as an example, K-ras is valid as biomarker in colon cancer but not in lung cancer for the detection of an inactive EGFR pathway in treatment with EGFR inhibitors.
- In the last example, potential drug targets (gene expression/genetics of EGFR, Her2) are estimated quantitatively. The drug effect on quantitative estimation is potentially contaminated by noise, what raises the issue of precision of estimate. Moreover, the efficacy depends on the overall quantitative amount of the target molecule and diagnostics relies on biopsy locus, time and tissue contribution.

**Table 3:** Examples of biomarker-drug pairs with comparative evidence

Pathogenetic mechanism	Examples Biomarker	Drug	Biomarker-efficacy relationship	Biomarker- safety relationship
Detection of the specific pathological change	BCR-Abl, EML4-ALK BrafV600E	Imatinib Crizotinib Vemurafenib	Efficacy zero if biomarker negative (expected to be predictive only)	Safety not dependent on biomarker
Measure of the activation of a pathway (yes–no)	FLT3 in AML K-ras	Crizotinib Cetuximab	Efficacy most probable if biomarker positive (expected to be predictive)	Safety not dependent on biomarker
Indirect measure of overexpression (dependent on sampling, cell heterogeneity)	EGFR, Her/neu, CD30	Erlotinib Trastuzumab brentuximab	Efficacy higher if biomarker positive (expected to be predictive and prognostic)	Biomarker-positive non-tumour cells may have safety implications

Some biomarker-drug pairs cannot be classified into categories D or E as its pharmacological comparative evidence (level C) suggests that the risk benefit becomes negative in BM- patients. Therefore, the generation of data in BM- patients needed for levels D and C may be unethical.

### Examples of Biomarker-Defined Drugs Approved by EMA

In the following, we present examples of 10 biomarker-drug pairs of 9 drugs for the proposed categories approved by the European Medicine Agency (EMA).

Identification of biomarker-defined drugs which are authorized in the EU is based on the PharmGKB Database [9]. Evidence for biomarker-defined subgroups are taken from documents published on http://www.ema.europa.eu like European Assessment Reports (EPAR) or SmPC [22–30].

Table 4 gives an overview of these biomarker-drug pairs classified in the proposed categories. The drugs’ indication is extracted from the drug labels published on the EMA homepage. Moreover, this table includes the biomarker’s data type, the type of outcome related to the biomarker-drug pair and the PGx label of PharmGKB referring to the genetic testing proposed in the drug labels of the EMA and US Food and Drug Administration (FDA).

**Table 4:** Examples of biomarker-drug pairs classified according to their evidence

Category proposed (Clinical endpoint)	Drug name/therapeutic area	Biomarker	PGx Level	Application in the EU	BM data type	Type of outcome
A	Belimumab (Benlysta)/Immunology-Rheumatology-Transplantation	TNFSF13B	*Informative* (FDA + EMA)	Indicated as add-on therapy in adult patients with active, autoantibody-positive systemic lupus erythematosus (SLE) with a high degree of disease activity	Binary	Efficacy
A	Pegloticase (Krystexxa)/Rheumatology	G6PD	*Testing required* (FDA + EMA)	Approved for treatment of severe debilitating chronic tophaceous gout in patients who may also have erosive joint involvement and who have failed to normalize serum uric acid with xanthine oxidase inhibitors at the maximum medically appropriate dose or for whom these medicines are contraindicated	Categorical	Metabolism
A	Eltrombopag (Revolade)/Purpura, Thrombocytopenic, Idiopathic	Factor V Leiden (F5 gene)	*Actionable(FDA* + *EMA)*	Approved to treat long-term immune (idiopathic) thrombocytopenic purpura and thrombocytopenia in patients with chronic hepatitis C. It is also used to treat acquired severe aplastic anaemia	Binary	Safety
A		And ATIII deficiency (SERPINC1)	*Actionable (FDA* + *EMA)*		Continuous	Safety
B (SVR rate)	Boceprevir (Victrelis)/Infectious Diseases	IL28B (IFNL3)	*Informative (FDA); Actionable (EMA)*	Approved for chronic hepatitis C (CHC) genotype 1 infection, in combination with peginterferon alfa and ribavirin, in adult patients with compensated liver disease who are previously untreated or who have failed previous therapy	Categorical	Efficacy
C	Osimertinib (Tagrisso)/ Oncology	EGFR T790M	*Testing required* (FDA + EMA)	Used for EGFR T790M mutations positive non-small cell lung cancer (NSCLC)	Binary	Efficacy
C	Eliglustat (Cerdelga)/ Inborn Errors of Metabolism	CYP2D6	*Testing required* (FDA + EMA)	Authorized for gaucher disease type 1 (GD1), who are CYP2D6 poor metabolizers (PMs), intermediate metabolizers (IMs) or extensive metabolizers. It should not be used in patients who are CYP2D6 ultra-rapid metabolizers (URMs) or indeterminate metabolizers	Categorical	Metabolism
D (response Rate and PFS)	Vandetanib (Caprelsa)/Oncology	RET	*Recommended (EMA)*	It is approved for aggressive and symptomatic medullary thyroid cancer (MTC) in patients with unresectable locally advanced or metastatic disease For patients with unknown or negative Transfection (RET) mutation a possible lower benefit should be taken into account	Categorical	Efficacy
D (mean area under the plasma concentration–time curve)	Esomeprazole (Nexium Control)/Gastroenterology	CYP2C19	*Actionable (FDA); Informative (EMA***)**	Authorized as short-term treatment of reflux symptoms (e.g. heartburn and acid regurgitation)	Continuous	Metabolism
D (PFS)	Ibrutinib (Imbruvica)/ Oncology	del (17p)	*Testing required* (FDA + EMA)	Amongst other indications approved for chronic lymphocytic leukaemia (CLL) with at least one prior therapy, or in first line in the presence of 17p deletion or TP53 mutation in patients unsuitable for chemo-immunotherapy	Binary	Safety

Table 4 gives examples of biomarker-drug pairs where comparative evidence either on a non-clinical level or both on clinical and non-clinical level is given (categories C and D). No biomarker-drugs pair could be identified yet, where sufficient confirmatory evidence (category E) is given to fully demonstrate biomarker-drug interaction on clinical endpoints. It is important to note that our proposal classifies the given evidence for biomarker-drug pairs. The available evidence on a biomarker-drug pair can change when further studies on biomarkers or biomarker-drug pairs are conducted. Therefore, the category a biomarker-drug pair is assigned to can change over time and has to be updated regularly.

For instance, osimertinib is classified into category C which means that only comparative pharmacological evidence is presented for the biomarker EGFR T790. Osimertinib is authorized for the treatment of advanced non-small cell lung cancer as first-line treatment for patients with activating epidermal growth factor receptor (EGFR) mutations or for patients with EGFR T790M mutations. Based on the EPAR [26] the inhibitory activity against EGFR was demonstrated in vitro. Tumour shrinkage in EGFRm and T790M NSCLC mouse lung tumour models was showed in vivo. However, the clinical studies for osimertinib did not include any patients without EGFR mutations. Therefore, osimertinib is classified into category C as EGFR is considered to be predictive for osimertinib based on pharmacological evidence, but it is not supported by clinical data.

An example for category D is vandetanib authorized for the treatment of medullary thyroid cancer [27]. For patients without Rearranged during transfection (RET) mutation the EMA drug label states a potential lower benefit of vandetanib compared to patients with RET mutation. Based on study 058 the hazard ratio (HR) of progression free survival (PFS) in the subgroups of RET mutation positive patients (*n* = 187) was 0.45 (95% CI 0.26–0.78), whereas in 79 patients proven without M918T mutation and with no other RET mutation the HR of PFS was 0.57 (95% CI 0.29–1.13), For vandetanib clinical data support the evidence of a differential treatment effect in patients with and without RET mutation and is therefore classified into category D.

## Summary and Discussion

Biomarkers used to define specific subpopulations of patients constitute the basis for a stratified or personalized medicine. In this context they are intended to predict either an improved treatment effect or a better safety profile in those patients who are defined by this biomarker. In contrast to prognostic biomarkers that indicate a favourable or unfavourable course of the disease, predictive biomarkers are capable to discriminate patient groups with different treatment effects.

Due to the potentially limited evidence on predictive biomarkers, difficulties in differentiating prognostic and predictive biomarkers and the possibility of chance findings in searches for biomarker-by-drug interactions in the presence of multiple options, it appears paramount to use a scheme that indicates increasing evidence for the usefulness of the biomarker.

In this paper we proposed a classification of biomarkers that is related to the mechanism of action, the relation to the drug target, and to the clinical evidence. The classification helps to differentiate levels of evidence and to assess whether current evidence is acceptable for drug-specific definitions of patient subgroups. The proposed classification could be used to strengthen and focus the discussion of novel biomarkers in the FDA’s and EMA’s biomarker qualification procedures and improve the comparability between different biomarker-drug pairs using clearer criteria. In addition, emerging new evidence (e.g. new clinical data in biomarker-defined subgroups) may be integrated by updating the initially proposed classification. Classification is based on the currently available evidence (with unclear evidence resulting in a lower classification) and could be upgraded in case new evidence is available. The proposed classification could be implemented in regulatory scientific advice to inform either further clinical development or the indication and product characteristics.

We classified biomarkers into five ascending categories with increasing evidence on the predictive nature of the biomarker in relation to a specific drug. Three subtypes are proposed to further describe the relation of the drug target. This classification is expected to support regulatory decision-making with respect to biomarker-related subgrouping, both, during clinical programmes and at the time of marketing authorization application. Whether a biomarker-related subgrouping is useful or not depends on the level of evidence.

In order to demonstrate the applicability of the proposed classification, we identified drugs with labels containing pharmacogenetic information. The corresponding biomarker-drug pairs approved by the European Medicine Agency were classified based on the proposed scheme. Typically, drugs classified in category C (biomarkers with comparative pharmacological evidence) were authorized in a biomarker-defined subgroup, only. We would like to encourage the collection of data in biomarker-negative patients to estimate the treatment effect in these patients as well unless prevented by ethical considerations. Nevertheless, these analyses do not require statistical significance; rather they are intended to substantiate stratification, refine the drugs indication and potentially improve benefit-risk. They could suggest, underpin or demonstrate a differential treatment effect, and hence support regulatory decision-making with respect to the definition of the patient population.

In other categories, empirical evidence is required. Here, studies using an adaptive enrichment design that adaptively select the population of interest can be used for efficient clinical development programmes [31, 32].

## References

[1] Tsourounis M, Stuart J, Pignato W (2015). Current trends in personalized medicine and companion diagnostics: a summary from the dia meeting on personalized medicine and companion diagnostics. Ther Innov Regul Sci..

[2] U. S. Food and Drug Administration, List of Cleared or Approved Companion Diagnostic Devices (In Vitro and Imaging Tools), https://www.fda.gov/medical-devices/in-vitro-diagnostics/list-cleared-or-approved-companion-diagnostic-devices-in-vitro-and-imaging-tools, September 2021.

[3] Biomarkers Definitions Working Group (2001). Biomarkers and surrogate endpoints: Preferred definitions and conceptual framework. Clin Pharmacol Ther.

[4] European Medicines Agency, Concept paper on predictive biomarker-based assay development in the context of drug development and lifecycle*.* Accessed 20 July 2017.

[5] European Medicines Agency, Reflection paper on co-development of pharmacogenomic biomarkers and Assays in the context of drug development. Accessed 24 Oct 2010.

[6] International Conference on Harmonization. International Conference on Harmonization (ICH) guidance, E9 Statistical Principles for Clinical Trials (ICH E9 guidance), 1998a. Available: https://www.ema.europa.eu/en/documents/scientific-guideline/ich-e-9-statistical-principles-clinical-trials-step-5_en.pdf.

[7] Senn S (2017). Mastering variation: variance components and personalised medicine. Stat Med.

[8] Wang S-J, Hung HMJ (2014). A regulatory perspective on essential considerations in design and analysis of subgroups when correctly classified. J Biopharm Stat.

[9] Firestein GS (2006). A biomarker by any other name…. Nat Clin Pract Rheumatol.

[10] Frank R, Hargreaves R (2003). Clinical biomarkers in drug discovery and development. Nat Rev Drug Discov.

[11] Walto MK Biomarkers and qualification: a focus on drug development. 2011. [Online]. Available: https://www2.rsna.org/re/QIBA_Annual_Meeting_2011/Index_files/WALTON.pdf. Accessed 19 Oct 2017.

[12] BEST (Biomarkers, EndpointS, and other Tools) Resource. FDA-NIH Biomarker Working Group. Silver Spring (MD): Food and Drug Administration (US); Bethesda (MD): National Institutes of Health (US); 2016. 27010052

[13] Whirl-Carrillo M, McDonagh E, Hebert J, Gong L, Sangkuhl K, Thorn C, Altman R, Klein T (2012). Pharmacogenomics knowledge for personalized medicine. Clin Pharmacol Ther.

[14] Vivot A, Boutron I, Ravaud P, Porcher R (2015). Guidance for pharmacogenomic biomarker testing in labels of FDA-approved drugs. Genet Med.

[15] Ansari M (2013). The regulation of companion diagnostics: a global perspective. Ther Innov Regul Sci..

[16] Enzmann H, Benda N, Meyer R, Scholl C, Stingl J, Broich K. Companion diagnostics and biomarker tests in the European medicines agency’s assessment of medicinal products. 2019.

[17] European Medicines Agency: EMA/446337/2011, Reflection paper on methodological issues with pharmacogenomic biomarkers in relation to clinical development and patient selection, Accessed 12 July 2011.

[18] European Medicines Agency: EMA/CHMP/SAWP/102001/2011. Qualification Opinion of Alzheimer’s Disease Novel Methodologies/biomarkers for BMS-708163. 2011.

[19] Guidance for Industry and FDA Staff Qualification Process for Drug Development Tools. http://www.fda.gov/downloads/Drugs/GuidanceComplianceRegulatoryInformation/Guidances/UCM230597.pdf. 2021.

[20] European Medicines Agency: EMEA/H/C/001016-Iressa: EPAR—Public assessment report 2009 https://www.ema.europa.eu/en/documents/assessment-report/iressa-epar-public-assessment-report_en.pdf. 2021.

[21] Hand DJ (1994). Deconstructing statistical questions. J R Stat Soc.

[22] European Medicines Agency: EMEA/H/C/002015- Benlysta: EPAR—Public assessment report 2011, https://www.ema.europa.eu/en/documents/assessment-report/benlysta-epar-public-assessment-report_en.pdf, 2021.

[23] European Medicines Agency: EMEA/H/C/002208-Krystexxa: EPAR—Public assessment report 2013, https://www.ema.europa.eu/en/documents/assessment-report/krystexxa-epar-public-assessment-report_en.pdf. 2021.

[24] European Medicines Agency EMEA/H/C/001110-Revolade: EPAR—Public assessment report 2010, https://www.ema.europa.eu/en/documents/assessment-report/revolade-epar-public-assessment-report_en.pdf. 2021.

[25] European Medicines Agency EMEA/H/C/002332-Victrelis: EPAR—Public assessment report 2011, https://www.ema.europa.eu/en/documents/assessment-report/victrelis-epar-public-assessment-report_en.pdf. 2021.

[26] European Medicines Agency EMEA/H/C/004124- Tagrisso: EPAR—Public assessment report 2016, https://www.ema.europa.eu/en/documents/assessment-report/tagrisso-epar-public-assessment-report_en.pdf. 2021.

[27] European Medicines Agency EMEA/H/C/003724-Cerdelga: EPAR—Public assessment report 2015, https://www.ema.europa.eu/en/documents/assessment-report/cerdelga-epar-public-assessment-report_en.pdf. 2021.

[28] European Medicines Agency EMEA/H/C/002315-Caprelsa: EPAR—Public assessment report 2012, https://www.ema.europa.eu/en/documents/assessment-report/caprelsa-epar-public-assessment-report_en.pdf. 2021.

[29] European Medicines Agency EMEA/H/C/002618-Nexium Control: EPAR—Public assessment report 2013, https://www.ema.europa.eu/documents/assessment-report/nexium-control-epar-public-assessment-report_en.pdf. 2021.

[30] European Medicines Agency EMEA/H/C/003791-Imbruvica: EPAR—Public assessment report 2014, https://www.ema.europa.eu/en/documents/assessment-report/imbruvica-epar-public-assessment-report_en.pdf. 2021.

[31] Maca J, Bhattacharya S, Dragalin V (2006). Adaptive seamless phase II/III designs—background, operational aspects, and examples. Ther Innov Regul Sci..

[32] Placzek M, Friede T (2019). A conditional error function approach for adaptive enrichment designs with continuous endpoints. Stat Med.

